# Pancreatic uptake and radiation dosimetry of 6-[^18^F]fluoro-L-DOPA from PET imaging studies in infants with congenital hyperinsulinism

**DOI:** 10.1371/journal.pone.0186340

**Published:** 2017-11-08

**Authors:** Pradeep K. Garg, Stephen J. Lokitz, Lisa Truong, Burton Putegnat, Courtney Reynolds, Larry Rodriguez, Rachid Nazih, Jonathan Nedrelow, Miguel de la Guardia, John K. Uffman, Sudha Garg, Paul S. Thornton

**Affiliations:** 1 Center for Molecular Imaging and Therapy, Biomedical Research Foundation, Shreveport, Louisiana, United States of America; 2 Cook Children’s Medical Center, Fort Worth, Texas, United States of America; Odense University Hospital, DENMARK

## Abstract

The aim of this study is to assess the radiation absorbed dose of ^18^F-Fluoro-L-DOPA derived from the Positron Emission Tomography (PET) images of infants age ranging from 2 weeks– 32 weeks and a median age of 4.84 weeks (Mean 10.0 ± 10.3 weeks) with congenital hyperinsulinism.

**Methods:**

After injecting 25.6 ± 8.8 MBq (0.7 ± 0.2 mCi) of ^18^F-Fluoro-L-DOPA intravenously, three static PET scans were acquired at 20, 30, and 40 min post injection in 3-D mode on 10 patients (6 male, 4 female) with congenital hyperinsulinism. Regions of interest (ROIs) were drawn over several organs visible in the reconstructed PET/CT images and time activity curves (TACs) were generated. Residence times were calculated using the TAC data. The radiation absorbed dose for the whole body was calculated by entering the residence times in the OLINDA/EXM 1.0 software.

**Results:**

The mean residence times for the ^18^F-Fluoro-L-DOPA in the liver, lungs, kidneys, muscles, and pancreas were 11.54 ± 2.84, 1.25 ± 0.38, 4.65 ± 0.97, 17.13 ± 2.62, and 0.89 ± 0.34 min, respectively. The mean effective dose equivalent for ^18^F-Fluoro-L-DOPA was 0.40 ± 0.04 mSv/MBq. The CT scan used for attenuation correction delivered an additional radiation dose of 5.7 mSv. The organs receiving the highest radiation absorbed dose from ^18^F-Fluoro-L-DOPA were the urinary bladder wall (2.76 ± 0.95 mGy/MBq), pancreas (0.87 ± 0.30 mGy/MBq), liver (0.34 ± 0.07 mGy/MBq), and kidneys (0.61 ± 0.11 mGy/MBq). The renal system was the primary route for the radioactivity clearance and excretion.

**Conclusions:**

The estimated radiation dose burden from ^18^F-Fluoro-L-DOPA is relatively modest to newborns.

## Introduction

^18^F-Fluoro-L-DOPA is a radiofluorinated analogue of the amino acid L-dihydroxyphenylalanine (L-DOPA). This compound plays a significant role in the study of dopaminergic function in the human brain using PET [1,2] especially for the objective measure of disease severity [3,4]. L-DOPA is a neutral amino acid analogue and in certain tissues it is converted to L-dopamine by the aromatic amino-acid decarboxylase (AADC) which enables its entry and accumulation in the catecholamine storage vesicles [5]. L-DOPA also accumulates in neuroendocrine cells and in pancreatic islet cells after conversion to L-dopamine through the action of AADC [6–8]. For such reasons, ^18^F-Fluoro-L-DOPA PET has found its role in assessing the altered AADC activity in pancreatic β-cell from increased insulin synthesis and hypersecretion of insulin by these cells in patients with congenital hyperinsulinism (CHI) [8–10]. Downstream impact of ADCC on L-DOPA metabolism and accumulation in various dopaminergic and neuroendocrine cells facilitated the use of, ^18^F-Fluoro-L-DOPA PET/CT in a wide array of clinical applications such as localization and staging of tumors [11, 12], targeting neuroendocrine tumors [13], targeting glomus tumors in patients with gene mutation [14], and diagnosis of Parkinson’s and other movement disorders [15]. More recently, there has been a renewed interest in utilizing ^18^F-Fluoro-L-DOPA PET to diagnose and define the extent of disease in infants with CHI [10, 16–18]. Despite added cost and radiation exposure from the use of ^18^F-Fluoro-L-DOPA PET in patients with CHI, many investigators including our group found the accuracy of ^18^F-Fluoro-L-DOPA PET in diagnosing focal form of the CHI [9,19,20]. In addition, accumulation pattern of ^18^F-Fluoro-L-DOPA further improved the surgical outcome in patients with CHI [21–23]. In addition to its accuracy in diagnosing focal form of CHI non-invasively, Additionally, the ^18^F-Fluoro-L-DOPA PET has been more accurate in differentiating focal and diffuse disease than invasive procedures such as trans-hepatic portal venous insulin sampling (THPVS) and arterial calcium stimulation with hepatic vein insulin sampling (ASVS) [24].

Since ^18^F-Fluoro-L-DOPA PET plays a unique role in a wide range of clinical studies, several investigators have reported on ^18^F-Fluoro-L-DOPA radiation dosimetry to ensure safe and careful use of this radiopharmaceutical. Since ^18^F-Fluoro-L-DOPA is mostly used in adult patients, the reported dosimetry data has been calculated from and modeled for a 70 kg adult [25–27]. Due to significant radiation dose to the bladder, initially it was recommended to limit the ^18^F-Fluoro-L-DOPA injection to < 74 MBq (2 mCi) in adults [27]. Subsequently a new imaging protocol introduced the voiding of urinary bladder contents at ~40 min post injection. Significantly lower exposure achieved from incorporating voiding intervals has allowed for a higher injection dose of ~ 333 MBq (9 mCi) [26]. There are no reported radiation dosimetry estimates generated directly from ^18^F-Fluoro-L-DOPA PET imaging of infants. The current radiation dosimetry data in the literature for infant dose has been calculated by modeling the data extracted from the PET scans performed in adults to an infant body mass model. Since PET scans in infants require conscious sedation, voiding is not an option, thus confounding the decision on choosing a safe injection dose in infants. Therefore, the aim of this study was to generate radiation dosimetry of ^18^F-Fluoro-L-DOPA directly from PET scans performed on infants. Herein, we report our results on the ^18^F-Fluoro-L-DOPA estimated radiation dose calculated from the PET scans performed on infants with hyperinsulinism.

## Materials and methods

### Subjects

This study was approved by the Radiation Safety Office and the Institutional Review Board of the Cook’s Children Health Care System. As per the institutional IRB instructions, written informed consent was obtained from the guardians of each participating infant prior to initiating the PET studies. Imaging data from a total of 10 infants (6 male, 4 female) with a median age of 4.84 weeks (ages range2 week to 32 weeks) and an average weight of 5.0 ± 1.7 kg (range 2.8–8.6 kg) are included in this study between January 2014 to January 2016.

### Synthesis of ^18^F-Fluoro-L-DOPA

All chemicals used in this synthesis were purchased from commercial suppliers such as Sigma chemicals (Sigma Aldrich, St. Louis, USA) or from ABx Advanced Biochemical (ABx, Radenberg, Germany). ^18^F-Fluoro-L-DOPA was produced using a previously reported three-step isotopic exchange method with minor modifications [28]. Briefly, a solution of (2S, 5S) *tert*-butyl 4-benzyloxy 2-fluoro 5-formylbenzyl) 2-*tert*-butyl 3-methyl 4-oxoimidazolodine 1-carboxylate (6 mg) in 800 uL of *N*, *N* dimethylformamide was added to a reaction vessel containing tetrabutylammonium ^18^F-fluoride. After heating the contents for 8 min at 80°C, 9 mL of water was added to the vial and the contents were passed through a C-18 Sep-Pak cartridge. The trap contents from the Sep-Pak were eluted with 1 mL acetonitrile into vessel 2. After evaporating the acetonitrile, a solution of meta-chloroperoxybenzoic acid (10 mg) in chloroform (1mL) was added and the vial was heated for 20 min at 60°C. Subsequent hydrolysis using 1 mL of hydrobromic acid (48% solution) followed by a semi-preparatory high-performance liquid chromatography (HPLC) purification provided the final product. The semi-preparatory HPLC was performed using Phenomenex C-18 Luna 5μ, 250x10 mm column that was eluted at a 4 mL/min flow with 1% acetic acid in water. The product peak from the semi-prep HPLC column was collected in a vial containing 4 mL saline and10μL of 0.1N NaOH. The vial contents were then passed through q 0.22 μ filter and into the final product vial. The radiochemical purity of the product was assessed on an analytical HPLC using a Phenomenex C-18 Luna 5μ, 250x4.6 mm column eluted at 1 mL/min flow and using the same buffer as for the semi-preparatory HPLC purification. The enantiomeric purity of ^18^F-Fluoro-L-DOPA was assessed using a Lichospher-100 RP-18, 5μ, 250x4.6 mm chiral column that was eluted at 1.5mL/min. The mobile phase for this assay was prepared by mixing 100 mL of 0.002M *N*, *N*-dimethyl-L-phenylalanine in methanol, 900 mL of 0.001M copper(II) acetate solution in water, and 200μL of acetic acid.

### PET imaging

The PET/CT images were acquired in HD mode on a Biograph-mCT PET/CT scanner (Siemens, Tennessee, USA) which consist of 4-rings and 22 cm field of view. The detailed description and imaging characteristics of this scanner have been described previously [29]. After the PET scan acquisition, the following method of reconstruction was used: iterative image reconstruction using a fully 3-D Ordinary Poisson Ordered Subset Expectation Maximization (3D OP OSEM) algorithm using HD-point-spread-function modeling [30] and with measured attenuation correction and a modeled scatter correction in addition to random correction using the delayed correction method [31]. Data acquired from image sets in counts/pixel were calibrated using the standard quantification procedures to report uptake values as Bq/cc or as standard uptake values (SUV).

Patients were NPO for 6 h prior to PET/CT scans. Blood glucose level was monitored and maintained between 60mg/dl and 75mg/dl during the entire procedure by titrating the glucose infusion rate. After positioning the patient on the scanner bed, conscious sedation was performed using Precedex with or without Propofol and constantly monitored by a neonatologist or an anesthesiologist for the duration of the procedure. ^18^F-Fluoro-L-DOPA was injected intravenously into the subject’s arm at a prescribed dose of 4.44 MBq/kg (Mean ± SD for 10 subjects: 25.6 ± 8.8 MBq; 0.7 ± 0.2 mCi). After acquiring a CT scan for attenuation correction purposes, three single-bed position PET scans (600 sec each) centered on the abdomen were acquired at 23.3 ± 4.0 min, 33.5 ± 4.0 min, and 47.0 ± 3.8 min post injection in 3-D mode. Two of the ten subjects had a fourth PET scan acquired at 57.0 ± 2.7 min post injection.

#### PET image analysis and calculation of residence time

Reconstructed axial, coronal and sagittal images were used to identify organs that were clearly delineated on the scans. Using PMOD 3.2 software (PMOD Technologies, Zurich, Switzerland), regions of interest (ROIs) were drawn manually for each of the image data-sets and across all image planes to obtain the amount of radioactivity in a given organ. All ROIs were drawn to minimize organ boundary edge artifacts and were cross-checked in coronal, sagittal, and axial directions to ensure no overlap among different ROIs. The ROIs were drawn on each axial slice, and were stacked subsequently to apply it to the whole-body image to render 3-D volume data. The CT images were utilized for the anatomical accuracy of the PET ROIs. The muscle uptake was calculated by multiplying the radioactivity contents in a thigh muscle ROI by a weighting factor as described below:

$$\%ID_{Muscle}=\%ID_{Thigh\;Muscle\;ROI}*\frac{Weight\;*\;\%muscle\;mass}{Vol_{Thigh\;Muscle\;ROI}*\rho_{muscle}}$$

The TACs were generated from the ROIs and total radioactivity in each organ at the individual PET scan time points is expressed as percentage of the injected dose (%ID) or percent injected dose/gram of tissue (%ID/g). Trapezoid integration was used to obtain the total amount of radioactivity accumulated in an organ between the first and the last PET scans. The accumulated radioactivity amounts between time zero (^18^F-Fluoro-L-DOPA injection time) and the first PET scan measured value was derived from the integration of an exponential function fitted to the PET scan measurements extrapolated from time zero to the first PET scan value (~ 20 min). We assumed that the radioactivity peaked by about 5 min post-injection with a linear rise for ^18^F-Fluoro-L-DOPA, as previously reported [25]. The radioactivity from the last PET scan measured value to infinity is assumed to deplete solely via physical decay [32]. The residence times were calculated as the numerical integration of the total radioactivity contents accumulated in a given organ from time zero to infinity.

#### Calculation of equivalent organ dose and effective dose

The absorbed radiation dose to various organs in infants was estimated by entering the residence time values into the OLINDA/EXM 1.0 software [33] and using the built-in ‘newborn model.’ The effective dose equivalent and the effective dose were determined using the methods published in the International Commission on Radiological Protection publication 30 and 60, respectively [34,35].

## Results

The ^18^F-Fluoro-L-DOPA was prepared in ~ 15% radiochemical yield and with >97% radiochemical purity. The overall synthesis time was ~105 min which included the HPLC purification step. The ^18^F-Fluoro-L-DOPA was eluted at ~9–11 min from the semi-preparatory HPLC column and at ~6–6.5 min from the analytical HPLC column. The product was reconstituted in saline for injection and sterile filtered using a 0.2 μ Millipore filter. The average specific activity (n = 10) of ^18^F-Fluoro-L-DOPA was 2.04 ± 0.34 GBq/μmol (55.14 ± 9.25 mCi/μmol) at the end of synthesis.

**Fig 1:**
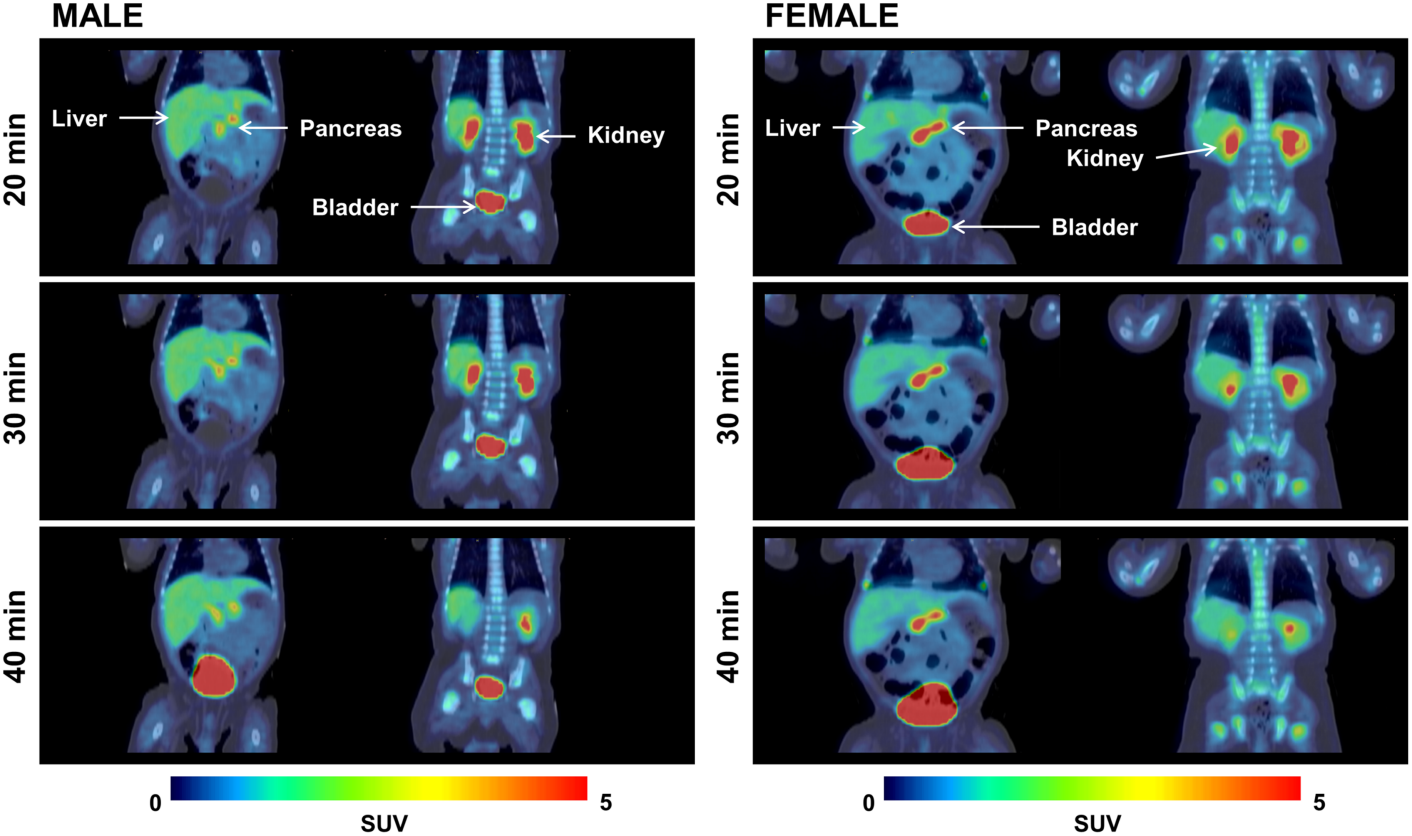
The uptake and distribution of ^18^F-fluoro-L-DOPA at 20, 30 and 40 min in a male and a female subject. Coronal views of representative PET images from a male (left) and a female subject (right). PET scans were acquired at ~20 min (top row), ~30 min (middle row), and ~40 min (bottom row) after injecting ^18^F-Fluoro-L-DOPA. For each row, the left image is an anterior view and the right image is the posterior view. Major organs such as kidneys, liver, pancreas and urinary bladder are clearly visible on early images (20 min). The uptake intensity in the organs decreases slightly with time, except for the urinary bladder.

Representative coronal views from ^18^F-Fluoro-L-DOPA PET images of a representative female and male subject are shown in Fig 1. A large ROI placed on the entire image acquired during the first PET scan contained ~60% of the administered radioactivity which is consistent with a single acquisition using a 22 CM field-of-view (FOV) scanner. Since single tomographic image set for this study covered only the abdominal area, the remainder of the radioactivity is assumed to be distributed in the rest of the body (body portion that remained outside the scanner FOV).

**Fig 2:**
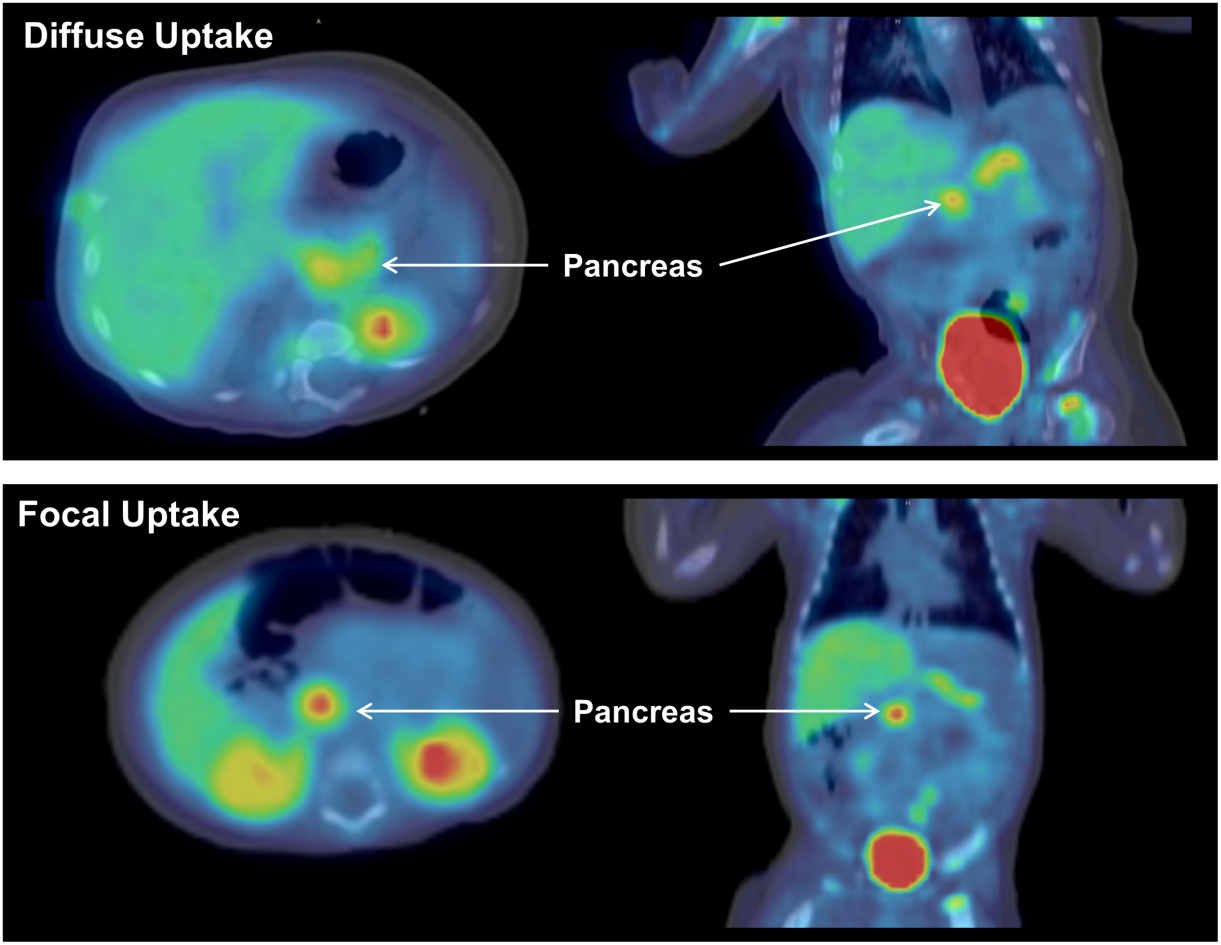
^18^F-fluoro-L-DOPA PET/CT scan showing the focal and diffuse uptake pattern in the pancreas axial (left) and coronal (right) view of ^18^F-Fluoro-L-DOPA scans from two representative subjects with hyperinsulinism. The top row shows a diffuse uptake of ^18^F-Fluoro-L-DOPA in the pancreas and the bottom row shows an intense focal accumulation of ^18^F-Fluoro-L-DOPA in the pancreas. Both the images were acquired at ~20 min post ^18^F-Fluoro-L-DOPA injection.

As shown in Fig 1, the radioactivity quickly accumulated in various tissues and several major organs were clearly delineated on the first PET image acquired at ~20 min. Accumulation of ^18^F-Fluoro-L-DOPA in the pancreas was also rapid and high, thus making it distinctly visible on 20 min scans (Figs 1 and 2). As anticipated, a significant amount of radioactivity accumulated in the urinary bladder and the radioactivity levels in the bladder continued to rise with time.

**Fig 3:**
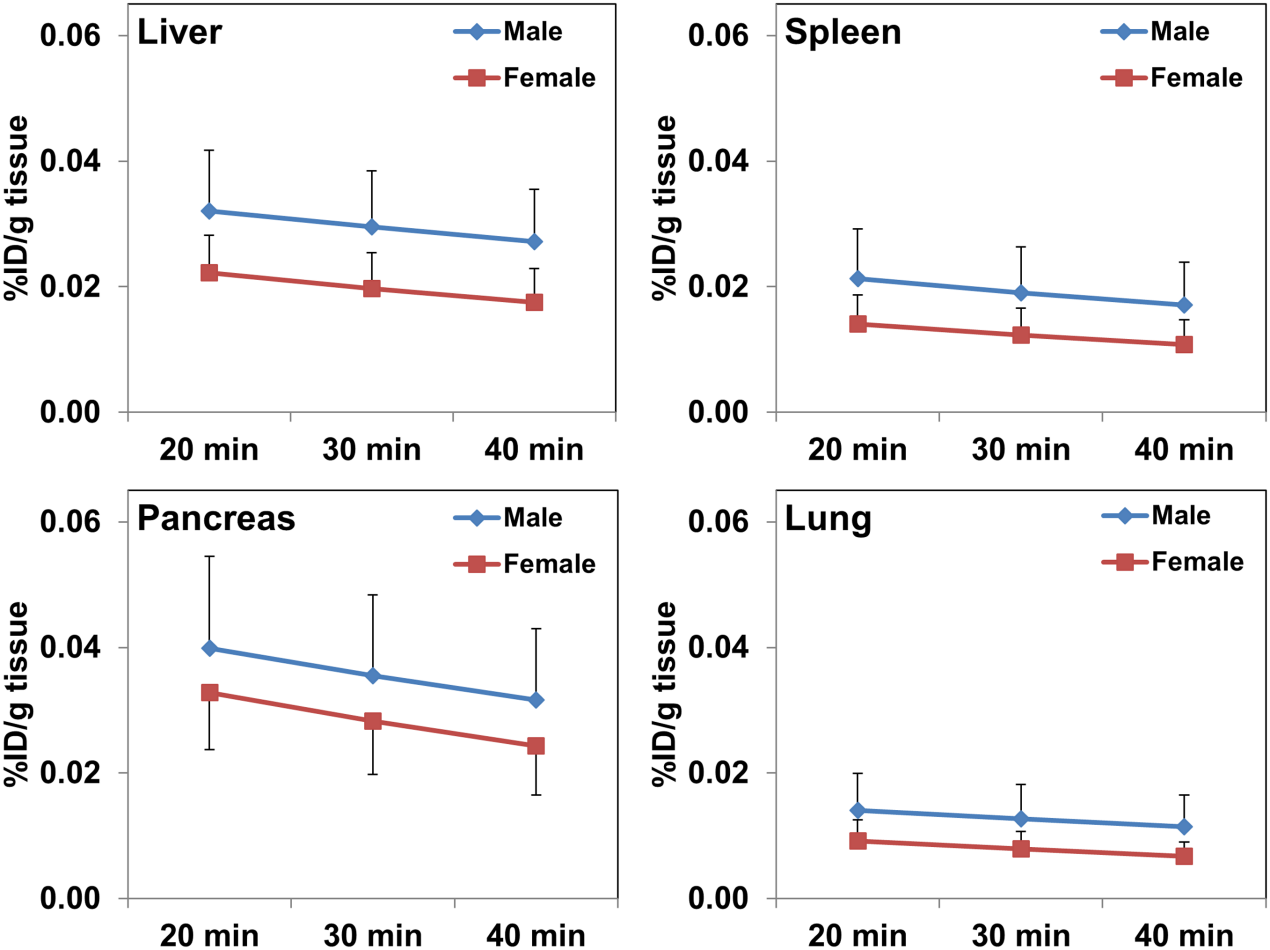
Mean values from time activity curves for organs easily delineable on ^18^F-Fluoro-L-DOPA PET scans from patients with hyperinsulinism (n = 10). While a slightly higher uptake of radioactivity is noted at all scan time points in male subjects, these differences were statistically insignificant (p>0.1). Radioactivity levels decreased marginally between 20 min and 40 min.

The TACs generated for the organs easily discernible on the PET/CT scans are presented in Fig 3. For most organs, a minimal washout of radioactivity was seen between the first scan and the last scan. For example, the %ID of F-18 in the liver, pancreas, lungs, and kidneys was 8.13 ± 1.65, 0.65 ± 0.24, 0.91 ± 0.26, and 3.96 ± 0.79, respectively, at ~23 min, and 6.49 ± 1.74, 0.48 ±0.18, 0.69 ± 0.21, and 1.98 ± 0.33, respectively, by ~47 min. As described earlier, the TACs generated for various organs from PET scan measured values and subsequently integrated from injection time to complete elimination of radioactivity were used to derive the residence times.

**Fig 4:**
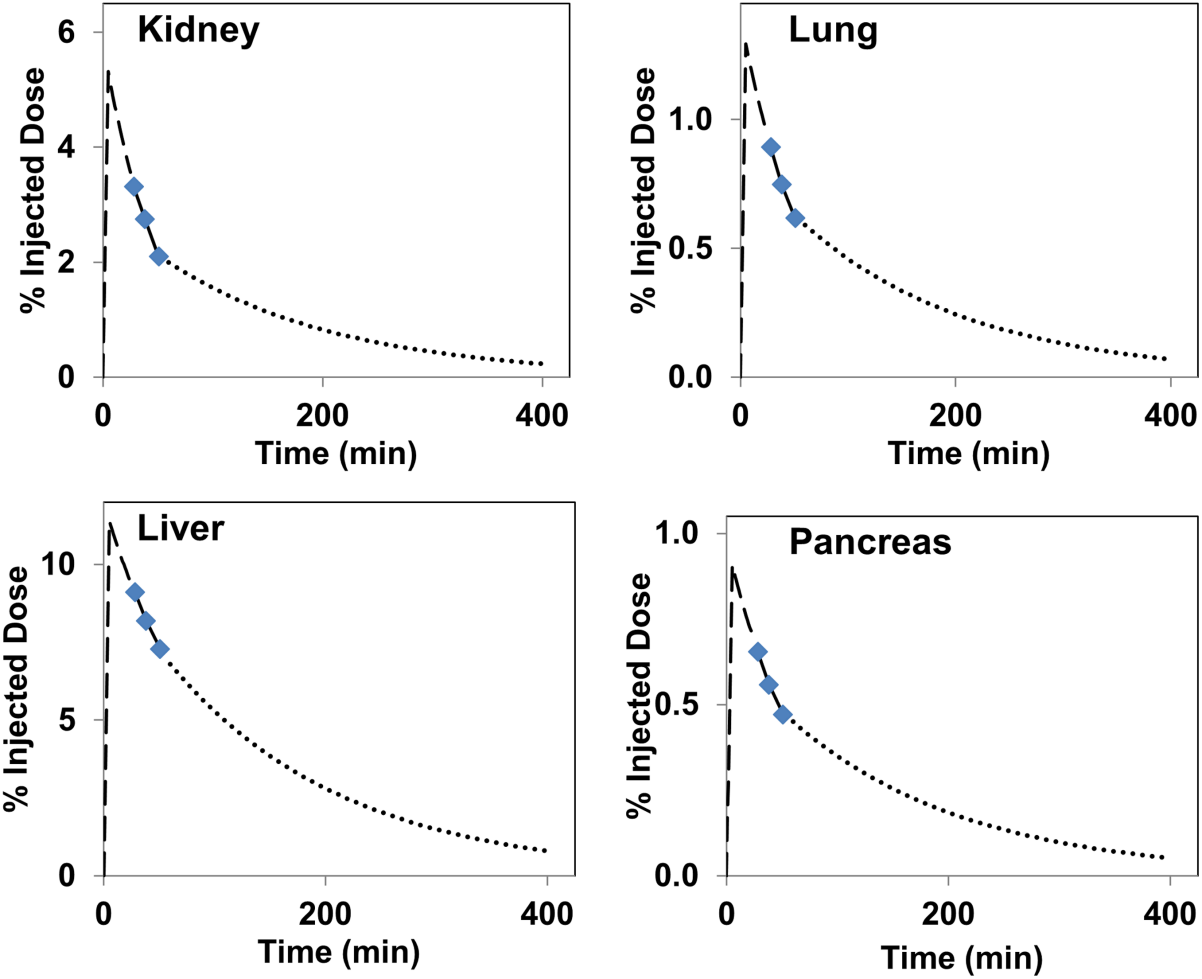
The time activity curves generated by fitting an expontial function to the PET data for the pancreas, liver, and kidneys. The initial portion of the curve (prior to 20 min PET scan value) was modeled to fit to a peak uptake at 5 min. The time activity curve past the 40 min scan measured value was assumed to be depleted only by the physical decay of F-18 radionuclide.

The TACs generated from extrapolating from time zero to infinity for select organs from representative PET scan is shown in Fig 4.

The mean residence times for various organs are presented in Table 1. No statistically significant differences in residence times were noted between the male and female organs.

**Table 1:** Residence times calculated from whole body^18^F-Fluoro-L-DOPA PET images acquired in newborns with hyperinsulinism.

Organ	Residence Time (min)	
	Male	Female
Liver	12.58 ± 3.00	9.97 ± 1.95
Lung	1.39 ± 0.30	1.03 ± 0.42
Bladder	19.75 ± 6.36	31.15 ± 8.17
Kidney	4.95 ± 0.98	4.20 ± 0.86
Muscle	17.51 ± 1.14	16.55 ± 4.20
Spleen	0.18 ± 0.11	0.25 ± 0.13
Stomach	0.13 ± 0.10	0.14 ± 0.02
Pancreas	0.86 ± 0.22	0.92 ± 0.51
Heart	1.10 ± 0.27	0.91 ± 0.04
Remainder of body*	**99.89 ± 2.64**	**93.24 ± 1.65**

* Male vs Female P<0.05

The radiation absorbed dose to various organs is shown in Table 2. The four organs that show the highest dose are the urinary bladder wall (2.76 ± 0.95 mGy/MBq), kidneys (0.60 ± 0.11 mGy/MBq), pancreas (0.87 ± 0.30 mGy/MBq), and the liver (0.34 ± 0.07 mGy/MBq). Based on the calculations from this study, the mean effective dose equivalent to a newborn would be 0.40 ± 0.04 mSv/MBq (1.49 ± 0.16 rem/mCi). As seen from Table 2, slightly higher effective dose equivalent values are noted for the females (0.44 ± 0.03 mSv/MBq) as compared to males (0.38 ± 0.03 mSv/MBq) and these differences are statistically significant (p<0.05). In addition to the radiation dose delivered from radiopharmaceutical, the calculated radiation dose from the CT scan performed for attenuation correction would be ~ 5.7 mSv [36]. Table 2 also compares the radiation absorbed dose obtained from studies performed in infants (this study) to data previously reported from studies performed in adults and modeled for 1-year old infant [37]

**Table 2:** Radiation dose estimates for ^18^F-Fluoro-L-DOPA in newborns (Mean ± SD).

*Organ*	*Dose (mGy/MBq)*	*Dose Rad/mCi*	^Ɣ^*Dose (mGy/MBq)*
Adrenals	0.16 + 0.01	0.58 ± 0.04	0.06
Brain	0.12 ± 0.00	0.44 ± 0.02	0.04
Breasts	0.11 ± 0.00	0.39 ± 0.02	0.04
Gallbladder wall	0.18 ± 0.01	0.65 ± 0.04	0.05
Lower large intestine wall	0.18 ± 0.01	0.68 ± 0.04	0.07
Small intestine	0.17 ± 0.00	0.63 ± 0.01	0.06
Stomach wall	0.16 ± 0.01	0.59 ± 0.05	0.05
Upper large intestine wall	0.17 ± 0.00	0.62 ± 0.01	0.06
Heart wall	0.13 ± 0.02	0.48 ± 0.07	0.05
Kidneys	0.60 ± 0.11	2.24 ± 0.42	0.14
Liver	0.34 ± 0.07	1.24 ± 0.26	0.05
Lungs	0.12 ± 0.02	0.44 ± 0.08	0.05
Muscle	0.12 ± 0.01	0.46 ± 0.03	0.05
Ovaries	0.19 ± 0.01	0.69 ± 0.04	0.07
Pancreas	0.87 ± 0.30	3.21 ± 1.11	0.06
Red marrow	0.22 ± 0.01	0.80 ± 0.03	0.04
Osteogenic cells	0.21 ± 0.01	0.78 ± 0.03	NR
Skin	0.11 ± 0.00	0.40 ± 0.01	0.04
Spleen	0.13 ± 0.03	0.49 ± 0.12	0.05
Testes	0.18 ± 0.02	0.66 ± 0.06	0.07
Thymus	0.12 ± 0.01	0.46 ± 0.02	0.05
Thyroid	0.13 ± 0.01	0.48 ± 0.02	0.05
Urinary bladder wall	2.76 ± 0.95	10.20 ± 3.51	1.00
Uterus	0.24 ± 0.03	0.90 ± 0.11	0.11
Total body	0.16 ± 0.00	0.60 ± 0.02	0.05
**Effective Dose Equivalent**	**0.40 ± 0.04 mSv/MBq**	**1.49 ± 0.16 rem/mCi**	
**Effective Dose**	**0.30 ± 0.04 mSv/MBq**	**1.12 ± 0.16 rem/mCi**	**0.10 mSv/MBq**

NR: Not reported

^Ɣ^: Data extrapolated to 1-year infant model from study performed in adults [37] and the data cited herein refers to the use of carbidopa for biokinetic calculations

## Discussion

L-DOPA is a large neutral amino acid that is a precursor to neurotransmitters such as dopamine, norepinephrine and epinephrine. After accumulation in specific tissues, amino-acid decarboxylase enzymes converts L-DOPA to L-dopamine, which is subsequently stored in the vesicles [38]. Preclinical studies show that L-DOPA accumulates and is then subsequently converted to L-dopamine in the pancreatic cells via the action of L-dopa decarboxylase enzymes [7]. ^18^F-Fluoro-L-DOPA is also a neutral amino-acid that biochemically resembles L-DOPA and similarly metabolizes [39]. Additionally, pancreatic β-cells show the presence of dopamine receptors [8] suggesting the rationale for selective and preferred targeting of these cells by ^18^F-Fluoro-L-DOPA. With its increased availability to clinical researchers and improved understanding of its biological distribution and accumulation pattern, the role of ^18^F-Fluoro-L-DOPA in newer clinical applications is growing. Therefore, ^18^F-Fluoro-L-DOPA has now found a wider acceptance in the clinic towards neurological as well as oncological applications. In addition, there is growing evidence on the usefulness of ^18^F-Fluoro-L-DOPA to assess primary hyperinsulinism in pediatric patients [10,16,23,40].

As one chooses the most effective targeting probe for PET imaging, it is important to have access to reliable radiation absorbed dose estimates for each patient population to ensure safe and effective use of that probe. Since ^18^F-Fluoro-L-DOPA has been in clinical use for a long time, there are multiple published reports on the radiation dosimetry of ^18^F-Fluoro-L-DOPA obtained through the whole body PET/CT imaging performed in adult subjects [25–27]. However, there is no published radiation dosimetry for ^18^F-Fluoro-L-DOPA calculated from PET scans performed on infants. Most radiation dosimetry estimates for children have been extrapolated from the data acquired from studies performed in adults. To our knowledge, this is the first report on radiation dosimetry estimates for ^18^F-Fluoro-L-DOPA derived from PET/CT imaging performed in infants with hyperinsulinism.

The ^18^F-Fluoro-L-DOPA was synthesized at our radiochemistry manufacturing site, ‘the Center for Molecular Imaging and Therapy’, located in Shreveport, LA. We prepared ^18^F-Fluoro-L-DOPA by using a slightly modified procedure of a previously published nucleophilic isotope exchange radiofluorination method [28]. In addition to the obvious advantage of a relatively simple synthesis procedure, the specific activity of the product prepared by this method is significantly higher than that synthesized via the electrophilic radiofluorination method [28]. The high radiochemical yields and adequate specific activity obtained through this method allowed us to transport ^18^F-Fluoro-L-DOPA to our distant imaging site at Cook’s Children Medical Center in Fort Worth, TX. Despite transporting ^18^F-Fluoro-L-DOPA to the PET imaging site four hours away, the average injected mass dose of ^18^F-Fluoro-L-DOPA at the time of injection was ≤ 3.5 μg/kg.

The injected dose of ^18^F-Fluoro-L-DOPA in these patients ranged between 3.7–7.3 MBq/kg body weight (0.10–0.20 mCi/kg) with an average total injected dose of 25.6 ± 8.8 MBq (0.7 ± 0.02 mCi). The injected dose was chosen based on our own previous experience and from reported dose used by others in infants with CHI [9,11,22,24]. Initially, we administered 3.7 MBq/kg (0.1 mCi/kg) and subsequently adjusted the injected quantity to 5.92 MBq/kg (0.16 mCi/kg). This modification was primarily intended to increase the overall count rate to help improve image quality, especially for the late image dataset. The quality of scans acquired from the initial (lower) injected amount and the higher injected quantity was remarkably similar. Therefore, we suggest an injection between 3.7 MBq/kg (0.10 mCi/kg) and 5.92 MBq/kg (0.16 mCi/kg) of body weight adequate for a good quality ^18^F-Fluoro-L-DOPA PET scans of infants. The uptake and accumulation of ^18^F-Fluoro-L-DOPA in the pancreas was rapid and provided clearly delineated images of the pancreas and several other organs on the PET/CT scan acquired at ~20 min post-injection (Fig 1). The ^18^F-Fluoro-L-DOPA uptake in the pancreas was noted as either a focal or diffuse type and is shown from representative scans for each type in Fig 2. While diffuse form of disease is difficult to treat, a focal lesion shown with PET scan would guide a limited pancreatic resection surgery which in turn minimizes the risk of diabetes mellitus [22]. To calculate the total accumulated radioactivity, the ROIs were drawn on the entire pancreas despite the gradation in the uptake pattern within this organ.

The TACs from various organs show that after an initial accumulation of radioactivity, these levels decrease slightly with time except for the bladder, where the radioactivity levels continue to rise due to continued accumulation through renal excretion (Fig 3). Although the muscles were not visible on the PET scans, the total radioactivity accumulated in muscles (%ID) was significantly higher than that in several of the distinctly visible organs on the PET images. Perhaps, the relatively high muscle mass (1.2 ± 0.4 kg) as compared to mass of the liver (~500g), pancreas (~20g), or the kidneys (~140g) is one of the contributing factors. The shape of time activity curves suggests that the peak uptake of ^18^F-Fluoro-L-DOPA occurred prior to the first PET/CT acquisition performed in this study. Despite low accumulation of radioactivity in normal tissues, several of the key organs including the pancreas were clearly visible on early PET images (Figs 1 and 2). Similar to earlier reports, renal excretion was seen as major route for radioactivity clearance in our study also [25,26].

The residence times calculated for various tissues agreed reasonably well across all subjects. The organs that were not visualized on the PET/CT images were pooled under ‘remainder of the body’. The maximum theoretical residence time [T_½_/ln(2) = 2.64 h for F-18] minus the sum of measured residence time obtained from discernible organs on PET images was assigned to the ‘remainder-of-the-body.’ These residence times were entered into the OLINDA software and the built-in newborn model [33] was used to obtain the radiation absorbed dose.

Although, the radiation dose estimates for ^18^F-Fluoro-L-DOPA from studies performed in newborns are not available for comparison, the dose estimates from ^18^F-FDG PET scan performed in newborns have been reported [41]. A head-on comparison of radiation absorbed dose from studies performed in infants show that ^18^F-Fluoro-L-DOPA delivers significantly lower radiation dose to newborns than that delivered from the ^18^F-FDG. For example, the radiation absorbed dose to the liver, spleen, lungs, heart wall, red-marrow, and kidneys was 0.41 ± 0.11mGy/MBq, 0.22 ± 0.05 mGy/MBq, 0.20 ± 0.05 mGy/MBq, 0.89 ± 0.26 mGy/MBq, 0.29 ± 0.08 mGy/MBq and 0.51 ± 0.14 mGy/MBq, respectively for the ^18^F-FDG and 0.34 ± 0.07 mGy/MBq, 0.13 ± 0.03 mGy/MBq, 0.12 ± 0.02 mGy/MBq, 0.13 ± 0.02 mGy/MBq, 0.22 ± 0.01 mGy/MBq, and 0.61 ± 0.11 mGy/MBq, respectively for ^18^F-Fluoro-L-DOPA. In general, most organs received a lower radiation dose per unit activity from ^18^F-Fluoro-L-DOPA administration than that from ^18^F-FDG. Additionally, radiation dose from attenuation correction CT scan is another source of exposure. The effective dose delivered from the attenuation CT scan depends on the acquisition parameters selected and it varies significantly between sites. It is important to recognize that radiation dose delivered from attenuation CT scan could be a significant portion of the total radiation dose delivered from the entire PET/CT procedure. For example, a 25 MBq injection of ^18^F-Fluoro-L-DOPA used in this study resulted in an effective radiation dose of ~7.55 mSv and the dose from CT attenuation scan (100kVp, dose modulating algorithm reference value of 218 mA and pitch 1.4) was ~5.7 mSv.

There is no reported dosimetry estimates available from PET studies performed using ^18^F-Fluoro-L-DOPA in infants. Nonetheless, the dosimetry estimates generated from studies performed in adults and data extrapolated to infants has been reported [25,26,37]. As shown in Table 2, significantly lower radiation absorbed dose per unit activity administered is reported when the data from studies performed in adults was extrapolated to infant-model. Among various possible factors for such differences, the use of carbidopa administration prior to PET scan to alter peripheral metabolism of ^18^F-Fluoro-L-DOPA and bladder voiding stand out as important factors that could influence the radioactivity distribution and excretion patterns [25]. In adult patients with hyperinsulinaemic hypoglycaemia (HH), pretreatment with carbidopa, a potent inhibitor of AADC, reduced the pancreatic uptake of ^18^F-Fluoro-L-DOPA within the normal pancreas which in turn assisted in delineating pancreatic tumor lesions, albeit only in half of that patient group [42]. In contrast, the use of carbidopa pretreatment in diagnosing infants with CHI show poor results and the use of carbidopa in patients with CHI remains controversial [43]. One of the factors responsible for diminished PET signal from pretreatment with AADC inhibitor carbidopa is disruption of ^18^F-Fluoro-L-DOPA decarboxylation step. This disruption triggering premature release of ^18^F-Fluoro-L-DOPA prior to its conversion to ^18^F-Fluoro-L-Dopamine and subsequent sequestration in vesicles via the amino acid transporters [43–45]

The results from this study show that the overall radiation exposure to infants from ^18^F-Fluoro-L-DOPA is modest. In addition, the studies show the benefit from inclusion of ^18^F-Fluoro-L-DOPA intervention to identify focal and diffuse CHI outweighs the associated risks. Limited field of view for PET scan performed in infants included in this study may be one of the limitation of this study. Nonetheless, in our data analysis to derive dosimetry, we assigned the radioactivity contents that were outside the FOV of the scanner to remainder of the body. Based on our results, we recommend injecting between 3.7 MBq/kg (0.10 mCi/kg) and 5.92 MBq/kg (0.16 mCi/kg) in infants and newborns for good quality ^18^F-Fluoro-L-DOPA PET scans.

## Conclusions

In conclusion, we evaluated the in vivo distribution and radiation dosimetry of ^18^F-Fluoro-L-DOPA derived from the PET/CT scans performed in newborns and infants with hyperinsulinism. The effective dose of ^18^F-Fluoro-L-DOPA in a newborn is 0.30 ± 0.04 mSv/MBq, suggesting a modest radiation dose to a newborn.

## Supporting information

S1 Table Supporting information includes age and gender of subjects, weight and injection dose. Accumulation of ^18^F-Fluoro-L-DOPA in key organs at various time points is presented as percent of injected dose per gram of tissue (%ID/g).
(XLSX)
